# Demography of a Eurasian lynx (*Lynx lynx*) population within a strictly protected area in Central Europe

**DOI:** 10.1038/s41598-021-99337-2

**Published:** 2021-10-06

**Authors:** Stefano Palmero, Elisa Belotti, Luděk Bufka, Martin Gahbauer, Christoph Heibl, Joe Premier, Kirsten Weingarth-Dachs, Marco Heurich

**Affiliations:** 1 Faculty of Environment and Natural Resources, University of Freiburg, Tennenbacher Straße 4, 79106 Freiburg, Germany; 2 Faculty of Forestry and Wood Sciences, Czech University of Life Sciences Prague, Kamýcká 1176, 16521 Prague 6, Czech Republic; 3 Department of Research and Nature Protection, Šumava National Park Administration, Sušická 399, 34192 Kašperské Hory, Czech Republic; 4 Department of Visitor Management and National Park Monitoring, Bavarian Forest National Park, Freyunger Str. 2, 94481 Grafenau, Germany; 5 Plant Biodiversity Research, Technische Universitӓt München, Emil-Ramann Straße 2, 85354 Freising-Weihenstephan, Germany; 6 Leibniz Institute for Zoo and Wildlife Research (IZW), Alfred-Kowalke-Str. 17, 10315 Berlin, Germany; 7 Inland Norway University of Applied Science Institute for Forest and Wildlife Management, Campus Evenstad, 2480 Koppang, Norway

**Keywords:** Zoology, Ecology

## Abstract

Large carnivores promote crucial ecosystem processes but are increasingly threatened by human persecution and habitat destruction. Successful conservation of this guild requires information on long-term population dynamics obtained through demographic surveys. We used camera traps to monitor Eurasian lynx between 2009 and 2018 in a strictly protected area in the Bohemian Forest Ecosystem, located in the core of the distribution of the Bohemian–Bavarian–Austrian lynx population. Thereby, we estimated sex-specific demographic parameters using spatial capture–recapture (SCR) models. Over 48,677 trap nights, we detected 65 unique lynx individuals. Density increased from 0.69 to 1.33 and from 1.09 to 2.35 individuals/100 km^2^ for open and closed population SCR models, respectively, with corresponding positive population growth rates (mean = 1.06). Estimated yearly sex-specific survival probabilities for the entire monitoring period were high (females 82%, males 90%) and per capita recruitment rate was low (females 12%, males 9%), indicating a low yearly population turnover. We ascertained an average number of recruits of 1.97 and a generation time of 2.64 years when considering resident reproducing females. We confirmed that reproduction in the study area took place successfully every year. Despite the overall increase in local lynx densities, the number of detected family groups remained constant throughout the study period. These results indicated that the strictly protected study area acts as a source for the multi-use landscapes in its surroundings. In this first open population SCR study on lynx, we provide sex-specific demographic parameters that are fundamental information for lynx management in the study area as well as in similar contexts Europe-wide.

## Introduction

Large carnivores shape ecosystems through top-down control of herbivores and intraguild predation of mesocarnivores, which in turn can trigger trophic cascades^1^. Their occurrence is therefore crucial for the functioning of ecosystems. However, large carnivore conservation is particularly challenging as carnivores’ food acquisition and large spatial requirements often lead them into conflict with human activities^2^ and their wide-ranging behaviour necessitate challenging transboundary management^3^. Moreover, low densities and reproduction rates make these species vulnerable to the effects of human persecution^4^. Consequently, a better understanding of the dynamics of large carnivore populations is imperative for successful conservation management^5^. Despite their importance, the temporal dynamics and environmental factors that drive populations are still poorly understood for many species. This is because only relatively few species are studied in the high detail needed for a thorough understanding. The necessity of sampling a large number of individuals over long periods makes it especially difficult to obtain a mechanistic understanding of why and how populations increase, decline, or go extinct. In most cases, even basic information, including demographic parameters such as abundance, density, survival and recruitment, are not available for populations of free-ranging animals, which makes conservation of threatened populations a hazardous game. Only a detailed time series of demographic parameters will allow a deeper understanding of population dynamics and reliable prediction of populations' future developments, which is crucial for well-informed conservation management. Therefore, long-term data collection is required^6^.

Information on demographic parameters is challenging to collect for large carnivores because they usually exhibit low population densities, are primarily nocturnal and often live in areas rich in cover. The rising popularity of camera traps for wildlife monitoring in recent years has helped to overcome some of these difficulties^7^. Moreover, such non-invasive devices allow simultaneous monitoring of different species and help to avoid stressful animal immobilisation^8^. Camera traps can provide high-quality pictures that enable the identification of naturally marked animals such as felids^9^. This has led to their extensive use in combination with capture–recapture (CR) methods for estimating demographic parameters of marked felids, e.g. tiger (*Panthera tigris*)^9^, ocelot (*Leopardus pardalis*)^10^, jaguar (*Panthera onca*)^11^ and various lynx species (*Lynx* sp.)^12^. Recently developed spatial capture–recapture (SCR) models offer improvements over conventional non-spatial CR models for estimating demographic parameters because they also incorporate spatial information such as the location of individuals and traps, and habitat suitability^13^. Closed population SCR models assume demographic closure, i.e. there is no emigration, immigration, mortality or reproduction, and are normally used to estimate abundance and density within one “session”^13^. More recent open population SCR models can be applied across multiple sessions, which has the advantage of providing further parameters, such as survival, per capita recruitment and population growth rate^14^, making them well suited for demographic analyses.

In the last century, the Eurasian lynx (*Lynx lynx*), was eradicated across Central Europe but, following legal protection and population reintroductions, the species has since recolonised parts of its former range^15^. However, most of the reintroduced populations in Central Europe have remained isolated and small, mainly due to human disturbance and habitat fragmentation^15^. As a typical example, the Bohemian–Bavarian–Austrian population, which was reintroduced in the 1980s and has been considered stagnating in recent years, is today classified as "endangered". This is despite the availability of sufficient suitable habitat^16^. From the perspective of a potential Central European metapopulation, the range of this population is located in a crucial area, but likely because of the low population size and spatial isolation, subadults cannot connect with neighbouring populations (e.g. the Harz, Carpathian, and Alpine)^17^, resulting in reduced genetic variability^18^. Illegal killing is considered as the mortality cause most constraining the Bohemian–Bavarian–Austrian population and the protected areas in the region are therefore crucial factors for its persistence^4^. The protection of source populations has been proposed as a strategy for recovering predator species, for example, the tiger (*Panthera tigris*)^19^. However, whether protected areas in Central Europe, including the study area, can host source populations with their limited size is poorly understood.

In this study, we conducted a 10-year (2009–2018) demographic study in the core of the distribution of a Eurasian lynx (hereafter lynx) population within one of Central Europe's largest strictly protected areas, the Bohemian Forest Ecosystem, using camera trapping and SCR methods. The results of this study help to improve our understanding of lynx demography in reintroduced populations. We used open population SCR models to estimate sex-specific demographic parameters such as abundance, density, survival probabilities, per capita recruitment rate and population growth rate. We expected higher density estimates for females since males have larger home ranges^20^. We estimated density via both closed and open population SCR models to compare the reliability of the different methods for assessing population status and allow cross-comparison with existing studies that used conventional closed population methods. As lynx is a K-selected species, we expected generally high survival probabilities and a low per capita recruitment rate. Specifically, we expected lower male survival probabilities because they generally take higher risks, for example by getting closer to human activities to exploit higher prey densities in those areas^21^ or patrolling their larger home ranges. Camera trapping data provided auxiliary information on reproductive parameters such as generation time and the average number of recruits, which we predicted to be at around 2 years of age and 1.5 kittens^22,23^. Finally, we calculated the relative abundance index (RAI)^24^ to obtain information on the development of lynx’s main prey and a common mesopredator.

## Material and methods

### Study area

**Figure 1:**
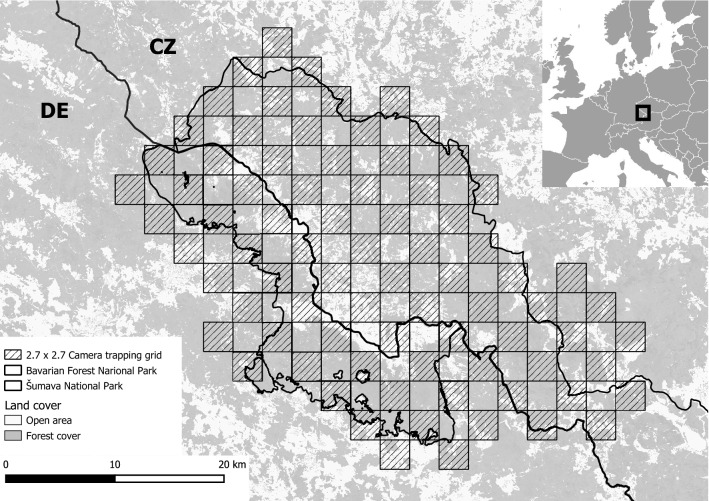
Map of the study area with forest coverage including the Bavarian Forest National Park (BFNP) on the German side (DE), the Šumava National Park (SNP) on the Czech side (CZ) and the 2.7 × 2.7 km camera trapping grid in which sites were located in every second cell. The map was created using QGIS 3.4 https://qgis.org/it/site/.

The Bohemian–Bavarian Forest is situated in Central Europe at the border between Austria, Czechia, and Germany, and includes two adjacent national parks: the Bavarian Forest National Park (BFNP) (240 km^2^) in Germany and the Šumava National Park (SNP) (690 km^2^) in Czechia (Fig. 1). These protected areas are not divided by ecological barriers and therefore represent a continuous area in the core of the Bohemian–Bavarian–Austrian lynx population distribution. The BFNP is surrounded by the Bavarian Forest Natural Park (3007 km^2^) and the SNP by the Šumava Protected Landscape Area (1000 km^2^), which together comprise the Bohemian Forest Ecosystem. Elevation ranges from 600 to 1456 m.a.s.l. and snow cover can persist for 5–8 months with greatest depths from January to March.

The area is covered by a mixed mountainous forest composed mainly of Norway Spruce (*Picea abies*), followed by European Beech (*Fagus sylvatica*) and Silver Fir (*Abies alba*)^25^ and hosts ungulate species such as roe deer (*Capreolus capreolus*), red deer (*Cervus elaphus*), wild boar (*Sus scrofa*) and moose (*Alces alces*)^26^. In the BFNP roe deer density ranges from 1.1 to 5 animals/km^2^^27^ and red deer was estimated at 1.56 animals/km^2^ via coordinated counting at winter feeding stations. Densities of both species are higher in the SNP^26^. Wildlife control within both national parks is conducted by trained staff and is limited to red deer and wild boar. Outside the national parks, roe deer, red deer and wild boar are hunted^26^.

The Bohemian-Bavarian-Austrian lynx population originates from 5 to 10 individuals illegally reintroduced in the 1970s and 17 individuals (11 males and 6 females) released officially in the 1980s^28^. The total population size, which includes the wide surroundings of our study area (13,000 km^2^), was recently estimated as 97–143 (mean = 120)^29^. Lynx density within the study area was estimated at 0.4–0.9 individuals/100 km^2^ in a preliminary study before the beginning of our study period^30^. Lynx is legally protected in all three countries but illegal killing still occurs^4^. The main prey species of lynx in the study area are roe deer and red deer with 80% and 17% of detected kills, respectively^31^.

### Camera trap monitoring

Data were collected between 2009 and 2018. The monitoring design was developed specifically for lynx but also provided data on other species. The spatial organisation of camera traps, developed by Weingarth et al.^30^, consisted of a 2.7 × 2.7 km grid with camera traps situated in every second grid-cell and positioned along forest paths, roads and trails (Fig. 1). The design maximises detection probability and avoids gaps that might include female home ranges, i.e. a minimum of 122 km^2^ estimated locally via radio-telemetry^16^. The camera trapping array occupied the entire BFNP and two-thirds of the SNP and measured 760 km^2^, i.e. almost twice the mean male home range size (436 km^2^) measured through telemetry^16^. This was in line with the camera trapping array size recommended to achieve adequate sample sizes for robust estimates^32^. Since individual identification requires high-quality pictures of both animal flanks, most camera trapping sites included two opposing cameras (Cuddeback) with white flash. In the BFNP, camera traps functioned almost constantly for 10 “lynx years”. A lynx year is defined as the period from 01/05 to 30/04 the subsequent year, since kittens are typically born on May^33^. In the SNP, most camera traps were only active from mid-September to the end of December and for technical reasons, camera trapping sites could not be active in year 2012. Camera trapping sites were sometimes slightly moved in the national parks at the beginning of each session and a few camera trapping sites were added in the study area over the entire monitoring period.

We identified lynx individuals from images by comparing their unique coat patterns. When the individual identification posed difficulties, pictures were judged by at least one additional expert with long term experience in this field. Poor quality images that precluded individual identification were discarded (~ 2% of captured images). Sex was determined by observing females with kittens or the genital area of the animal. Age could only be assessed for individuals first photographed as kittens and recaptured over the years. Additionally, we followed Weingarth et al.^29^ and assigned animals into two categories referring to their status: "juvenile" and "independent". The juvenile category included individuals < 1-year-old, i.e. kittens detected with their mother. Individuals in this category were excluded from the SCR analysis because of the high mortality of kittens^34^. The independent category consisted of all individuals > 1-year-old (i.e. subadults and adults) and individuals of unknown age but with proof of independence. This category included floaters (non-resident or dispersing individuals), which were defined either as known juveniles of the previous year, in their second year of life, or as individuals of unknown origin that appeared for the first time in the study area whose age could not be determined. In both cases, these individuals held no territories and could either disperse from or settle in, the study area the following year. All individuals of unknown status were discarded from analyses.

### Spatial capture–recapture models

Demography of lynx was investigated using both open and closed population SCR modelling frameworks. Although the former method allows estimation of the full range of demographic parameters, we included the latter for backward compatibility with previous studies on lynx conducted in Europe using closed population SCR models^35,36^, as well as for comparison of both methods within one study.

Following Weingarth et al.^37^, we selected a time frame of 100 days from 15/09 to 24/12 of each year as a primary period to ensure demographic closure and a sufficient number of recaptures for robust estimates. The first primary periods were not placed in such an ideal period. Specifically, camera traps started functioning in November in both the two national parks in year 2009 and just in the SNP in year 2010 and 2011. In the fourth primary period (i.e. year 2012), as already mentioned, camera traps were not active in the SNP. We combined all primary periods according to a classical "robust design"^38^, and thus performed open population SCR analysis. We defined one secondary period as one day and restricted the number of detections to at most one per site in any secondary period in line with a Bernoulli distribution, thereby reducing temporal autocorrelation. As such, our study included ten primary periods with 100 secondary periods each. The SCR method assumes the baseline detection probability *g*_*0*_ of any individual declines with the distance from its theoretical home range centre, the detection function scale *σ*, in the state space *S*, which should be at least 3*σ*,^13^. We, therefore, created a rectangular state-space mask with a continuous buffer of 18,000 km^2^ around camera traps based on results of preliminary closed SCR analyses (i.e. *σ* ~ 3.5–4 km). We used a density-independent population growth model adapted for sex-specific demographic parameters^14^. The population growth model was based on a spatial point process indicating the number and location of individuals at the initial population state (*t* = 1) and modelling abundance and distribution at the time *t* = 2 as a function of both survival probabilities and per capita recruitment rate. Using the R package “OpenPopSCR”^39^, we ran ten pooled chains comprising 100,000 Markov chain Monte Carlo iterations each with an augmented observed population size *M* of 400 individuals, i.e. much greater than the overall number of independent individuals detected across all primary periods. We estimated combined and sex-specific yearly abundance and density by dividing abundance by the area of the state space. We used a spatially explicit movement model to estimate sex-specific yearly survival probabilities and per capita recruitment rate separately from emigration and immigration, respectively^40^. For movement, we used a *Markov* activity centre relocation type^41^ with activity centres of individuals in primary period *l* + 1 centred around the activity centre in the first primary period *l* in the state-space according to a bivariate normal distribution. Estimates of per capita recruitment rate indicated the number of individuals per sex class added to total abundance each year. Realised sex-specific yearly population growth rate and sex ratio were estimated as derived parameters. The former was derived from the sex-specific abundances. The sex ratio indicated the probability of any individual being a female. Unknown sexes were considered as a latent covariate and estimated through the sex vector augmented to the length of *M*. The movement parameter indicated yearly activity centre relocation according to the *Markov* activity centre relocation type. All point estimates were obtained using posterior modes and interval estimates were calculated through 95% highest posterior density (HPD) intervals at the 2.5% and 97.5% quantiles of the posterior distribution^13^. We used the Gelman-Rubin statistic to assess convergence of open population SCR parameters through the “gelman.diag” function from the R package “coda”^42^. This calculates the potential scale reduction factor for each parameter with upper confidence limits (CI), whereby parameters with a 95% upper CI substantially above 1 are considered to lack convergence^43^.

Closed population SCR models in a maximum likelihood framework were fitted using the R package “secr”^44^. We defined one sampling occasion as 5 days^9^ and used detector type *proximity*^44^ according to a Bernoulli distribution to estimate the combined density of males and females. To allow method comparison, we used for each session a continuous buffer of 18,000 km^2^ as for open population SCR models and created rectangular state-space masks accordingly (Supplementary Figs. S1–S10). First, we fitted a model *M*_*0*_ which assumes baseline detection probability *g*_*0*_ and detection function scale *σ* to be equal for all individuals^45^. Secondly, we included sex as a covariate for both parameters and fitted two different models for each session, i.e. one keeping baseline detection probability *g*_*0*_ constant and testing sex on the detection function scale *σ* and one testing sex on both parameters for each session. We compared Akaike's Information Criterion corrected for small sample sizes (AICc) to determine the best model for our data and kept those with ΔAICc < 2 for model-averaging^46^. Uknown sexes were considered as a latent covariate by performing a hybrid mixture model^44^. This model consists of a combination of latent (e.g. missing sexes) and known classes (e.g. male and female) and estimates the mixing proportion of the different classes by calculation of the parameter “pmix”, which corresponds to the sex ratio. Additionally, this parameter allows class-specific modelling of the detection parameters for investigating sex differences, for example, in the detection probability. Statistical significance was evaluated using the 95% CI.

### Reproductive parameters and age distribution

Based on camera trapping data, we investigated reproductive parameters such as generation time and number of recruits. The first was calculated as the mean age of resident reproducing females (i.e. females with kittens) at their first documented reproduction. Age was assessed only for individuals first photographed as kittens and recaptured over time. We, therefore, only included resident reproducing females of known age in the calculation. In contrast, no resident reproducing females were discarded when calculating the average number of recruits. This was calculated as the overall mean number of kittens with the mother at the onset of winter (November–January)^21^.

Finally, we also included all the independent individuals of known age and sex detected during the primary periods for investigating the age distribution of the population.

### Relative abundance index

When species cannot be individually identified, camera trapping data can be used to calculate abundance indices^47^. The relative abundance index (RAI)^23^, which expresses the number of captures per monitoring day, is widely used in wildlife research^47^.

We matched the session length for RAI to that of the SCR analysis and considered lynx's main prey species, i.e. roe deer and red deer, and red fox (*Vulpes vulpes*). To avoid temporal autocorrelation, we defined capture events with a threshold of 10 min^48^ and bootstrapped the RAI estimates by randomly selecting one camera trap per site at a time over 5000 replicates to reduce camera and site capture biases. This aspect of the study was conducted in the BFNP only.

### Use of experimental animals

No animals were used or handled in the study.

## Results

### Camera trap monitoring

We detected 65 unique independent individuals (25 males, 28 females and 12 individuals of unknown sex) in a total of 48,677 trap nights (Table 1). For the reasons mentioned in the methods, the standardised session length of 100 days was not achieved in the first three primary periods (i.e. year 2009, 2010 and 2011) and in the fourth primary period (i.e. year 2012) camera traps were not active in the SNP, potentially resulting in estimates with higher uncertainty. The overall number of trapping nights was partially reduced during each season by technical problems such as camera trap failure, theft or snowfall. Considering the primary periods, only ten pictures (between 0 and 2% depending on the primary period) did not allow individual identification because of poor quality, while the remaining pictures were identified with certainty. We observed an increase in numbers of juveniles and independent individuals including males, females and individuals of unknown sex, while the number of resident reproducing females (i.e. females with kittens) remained quite stable. We could determine the number of floaters in most years, as well as their origin. Around 33% of the detected floaters were born in the study area while around 67% were born outside or had unknown origin. Compared to the other categories of independent lynx, the number of floaters was the value that fluctuated the most (from 0 in session 2013 to 10 in session 2017, see Table 1). Apart from three and four individuals in the first and third sessions, respectively, the status (i.e. juvenile or independent) was determined. The number of unique lynx events (at most one detection per site in any secondary period) including independent individuals increased by around six times over the monitoring period. For the German side, we were able to confirm human-related mortality causes of some detected individuals in the vicinity of the study area, which showed an increase in traffic accidents and occasional illegal killing. In particular, three adults, two subadults and two juveniles were killed in traffic accidents in the BFNP, while two adults and one juvenile of unknown sex were killed illegally outside the national park in Germany.

**Table 1:** Summary of the lynx information obtained every primary period, including: the number of independent females and resident reproducing females (with kittens), males, individuals of unknown sex, total number of independent individuals including floaters (– indicates not available), juveniles, individuals of unknown status, camera trapping sites, effective trapping nights, unique lynx events (at most one detection per site in any secondary period) of independent individuals and documented mortality cases with known causes.

Session	Status						Camera trapping sites	Effective trapping nights	Unique lynx events	Known mortality causes (German side only)	
	Independent				Juvenile^b^	Unknown					
	Female (with kittens)	Male	Unknown sex	Total (floaters)						Traffic accidents	Illegal killing
2009^a^	6 (4)	3	0	9 (–)	8	3	55	1728	23	0	0
2010^a^	6 (4)	6	1	13 (4)	8	0	62	3813	52	0	0
2011^a^	8 (3)	7	1	16 (7)	5	4	66	4124	82	0	0
2012^a^	7 (3)	5	1	13 (5)	5	0	31	2621	48	0	1
2013	8 (6)	7	1	16 (0)	8	0	66	5885	86	0	1
2014	9 (6)	8	0	18 (2)	9	0	65	6151	94	1	0
2015	11 (2)	10	0	22 (6)	5	0	64	6022	135	1	1
2016	12 (5)	11	3	27 (6)	11	0	65	5964	144	2	0
2017	11 (4)	14	3	29 (10)	6	0	69	5957	121	2	0
2018	11 (7)	13	1	25 (4)	15	0	69	6412	130	1	0

^a^The first three sessions had reduced number of effective trap nights because the monitoring started later in November in both the national parks in year 2009 and just in the SNP in years 2010 and 2011. For the session 2012, due to technical reasons, lynx camera trapping only took place on the German side of the study area.

^b^The number of juveniles referred to the entire lynx year.

### Spatial capture–recapture models

**Figure 2:**
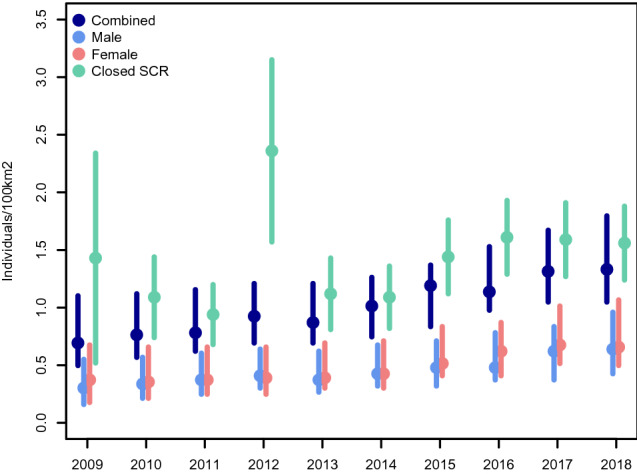
Posterior modes and 95% highest posterior density (HPD) intervals of open population spatial capture–recapture (SCR) models referring to combined, male and female lynx densities.

The combined density estimates for each primary period using open population SCR models increased from 0.69 (95% HPD intervals 0.50–1.10) to 1.33 (95% HPD intervals 1.05–1.79) individuals/100 km^2^ while, as expected, sex-specific density estimates were on average higher for females (Fig. 2). However, the overlapping 95% HPD intervals suggest there was no statistically significant difference between sexes. Combined abundance ranged from 38 (95% HPD intervals 26–60) to 75 (95% HPD intervals 58–100) individuals, that of males from 17 (95% HPD intervals 9–31) to 36 (95% HPD intervals 21–50) and that of females from 21 (95% HPD intervals 10–38) to 37 (95% HPD intervals 26–58). Concerning closed population SCR models, estimates of density ranged from 1.09 (*SE* 0.35) to 2.36 (*SE* 0.79) individuals/100 km^2^ (Table 1; Supplementary Table S2) and fluctuated strongly compared to open population SCR models (Fig. 2). Abundance ranged from 47.94 (*SE* 11.03) to 121.25 (*SE* 42.45) (Supplementary Table S2). Density estimates from each method fell just within the respective 95% CI or HPD of the other method, apart from the year 2012, indicating estimates were comparable and suggesting no statistically significant difference across methods. Further results of closed population SCR models are shown in the appendix (Supplementary Tables S2, S3).

All Gelman–Rubin diagnostic statistics had a 95% upper CI < 1.1 indicating convergence was reached. The parameter posterior modes (Table 2) of the yearly baseline detection probability *g*_*0*_ were equal across sexes. The yearly per capita recruitment rate was higher for females. The yearly survival probabilities of males, i.e. 0.90 (95% HPD intervals 0.79–0.96), were higher than those of females, i.e. 0.82 (95% HPD intervals 0.72–0.91). Males showed a higher yearly detection function scale *σ*, due to larger home ranges. However, considering the overlap of 95% HPD intervals, none of the parameters suggested a statistically significant difference across sexes. The realised population growth rate was 1.06 (range 1.01–1.10), that of males was 1.07 (range 1.00–1.12) and that of females was 1.05 (range 1.00–1.14), suggesting there was no predominant sex in the population. The probability of any individual to be a female was quite stable over years ranging from 0.51 (95% HPD intervals 0.36–0.65) to 0.56 (95% HPD intervals 0.33–0.76), meaning the sex ratio was slightly skewed towards females, with, however, no apparent statistically significant difference.

**Table 2:** Posterior modes and 95% highest posterior density (HPD) intervals of open population spatial capture–recapture (SCR) model referring to male (M) and female (F) lynx yearly baseline detection probability *g*_*0*_, detection function scale *σ* (km), survival probabilities and per capita recruitment rate.

Parameter	Sex	Estimate	95% HPD intervals	
			Lower	Upper
Detection probability	M	0.01	0.01	0.02
	F	0.01	0.01	0.01
Detection function scale	M	4.07	3.80	4.38
	F	3.96	3.66	4.32
Survival probabilities	M	0.90	0.79	0.96
	F	0.82	0.72	0.91
Per capita recruitment rate	M	0.09	0.04	0.14
	F	0.12	0.07	0.20
Movement	C	4.75	4.03	5.65

Per capita recruitment rate indicates the number of individuals of each sex added per year per total abundance. The yearly movement (km) was calculated for sexes combined (C).

### Reproductive parameters and age distribution

Regarding reproductive parameters, we included 14 resident reproducing females of known age and calculated a mean generation time of 2.64 years. One individual was first detected with kittens when it was seven years old and thus considered an outlier. The average number of recruits was 1.97 (range 1–3) and was calculated from information on 22 detected females (Supplementary Table S4).

Age and sex could be confirmed for a total of 24 independent individuals (15 females and 9 males) and were included in the age distribution pyramid (Supplementary Fig. S11). The oldest individual of known age was a 10-year-old male individual. The number of females at each age was equal to or higher than that of males except for ages 8–10.

### Relative abundance index

**Figure 3:**
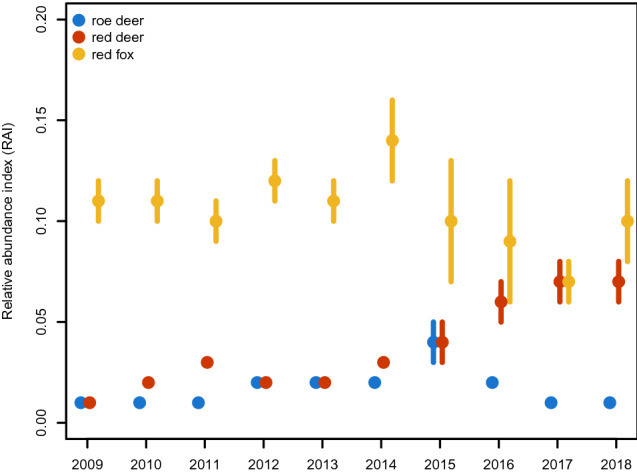
Estimates and standard deviations of the relative abundance index (RAI) of red fox and lynx's prey species roe deer and red deer calculated for the BFNP only.

The number of camera trapping sites used to calculate the RAIs ranged from 29 to 31 and effective trapping nights was 26,335 (Table 3). The RAI of red fox ranged from 0.07 (*SD* 0.01) to 0.14 (SD 0.02), that of red deer from 0.01 (*SD* 0.00) to 0.07 (*SD* 0.01), that of roe deer from 0.01 (*SD* 0.00) to 0.04 (*SD* 0.01) (Fig. 3). Fox abundance increased from 2009 to 2014 and decreased in the remaining years, overall showing an oscillating trend. Red deer and roe deer slightly increased until 2015, after which the former almost doubled while the latter decreased to earlier values.

**Table 3:** Summary of the available information about roe deer, red deer and red fox obtained every primary period, including number of events, camera trapping sites and effective trapping nights.

Session	Events			Camera trapping sites	Effective trapping nights
	Roe deer	Red deer	Red fox		
2009	12	12	136	29	1238
2010	28	56	306	31	2786
2011	29	87	291	31	2914
2012	52	52	315	31	2621
2013	57	57	315	31	2865
2014	57	86	401	30	2865
2015	110	110	274	29	2745
2016	57	172	58	29	2871
2017	27	189	189	29	2698
2018	27	191	273	29	2732

The number of events refers to the mean number of events resulting from all iterations in which one camera trap per site was sampled at a time.

## Discussion

Our modelling approach revealed that the number of independent lynx detected in the Bohemian–Bavarian Forest has increased over the past decade, with concurrently increasing abundance, density and positive population growth rates. Survival probabilities were high and per capita recruitment rate was low indicating a low yearly population turnover. Reproductive parameters such as generation time and average litter size considering all resident reproducing females indicated successful reproduction occurred every year, and the number of reproducing females remained stable throughout the study period despite the increase in the total number of detected independents. All these findings indicate that the protected areas act as an important, stable source area for the Bohemian–Bavarian–Austrian lynx population.

### Comparison of spatial capture–recapture methods

To our knowledge, this is the first demographic study on lynx comprising annual camera trapping over a decade and using open population SCR models. These models can provide a wide range of demographic parameters useful for long-term lynx monitoring. In addition, estimates from open population SCR models were not prone to inter-annual fluctuations that affected closed population SCR models and were generally lower, due to lower abundances compared to those from the latter (Table 1; Supplementary Table S2) divided by an equal buffer area. However, higher estimates with greater uncertainty, concerning the years 2009 and 2012, for which data were least complete, resulted from issues when accounting for varying efforts, i.e. the exact number of days the cameras were working. Allowing for varying effort has the benefit that detection parameters are related to a specific unit of effort thus being unbiased^44^. In the open population SCR models, the activity of each camera trap was informed over the primary periods. Concerning closed population models, in the year 2009, it was not possible to include the period from September to November because no cameras were functioning, resulting in zero occasions. While in the year 2012, the activity of the cameras in the SNP could not be specified because the devices were not in the field in the yearly camera trapping array that year. Therefore, data gaps resulted in biased estimates in this case because some spatio-temporal requirements, namely adequate sample size and number of recaptures, were not fulfilled. Closed population SCR density estimates of primary periods for which it was possible to account for varying effort, including years 2010 and 2011 for which data were also not complete, were comparable to those from open population SCR models since their 95% CI and HPD intervals overlapped. One advantageous feature of open population SCR methods in a Bayesian framework is their ability to deal with incomplete detection (e.g. non-annual monitoring). This is possible because they produce posterior distributions of the demographic parameters that incorporate the uncertainty resulting from data gaps by using information derived from other primary periods^14^. This highlights the robustness of this method, which we recommend for future studies and monitoring, especially when incomplete detections occur^49^. Nonetheless, closed population SCR models still represent a reliable and conventional tool for abundance and density assessments^35^.

### Spatial capture–recapture models

Our density estimates of both open (0.69–1.33 individuals/100 km^2^) and closed population SCR models (1.02–2.39 individuals/100 km^2^) were on the same order of magnitude as those resulting from other closed population SCR studies conducted on both reintroduced and autochthone populations across Europe. For example, the reintroduced populations of the French Jura and Vosges Mountains, with the lowest density estimate reported in Europe (0.24–0.91 individuals/100 km^2^)^35^, or the Swiss Alps (1.47 and 1.38 individuals/100 km^2^)^36^, and the autochthone population of the Western Carpathians (0.26–1.85 individuals/100 km^2^ of suitable habitat)^50^. The only outlier is a subpopulation in southwest Asia (Turkey) with a high lynx density of 4.20 individuals/100 km^2^^51^. However, lynx in Turkey live under different ecological conditions compared to those in Western and Central Europe, feeding mainly on lagomorphs instead of ungulates which leads to smaller home range sizes^52^.

In our study, the yearly baseline detection probability *g*_*0*_ was equal between sexes (Table 2). Although males are generally more active in order to patrol their larger home ranges, females with kittens hunt at a higher rate, which can result in augmented activity^53^, and might explain similarities across sexes for this parameter. The yearly detection function scale *σ* was not significantly higher for males compared to females (Table 2). This suggests similar home range sizes, which stands in contrast to expectations for lynx^20^ and most felids^10^. However, the restricted and seasonal session period was chosen for demographic closure and not appropriate for annual home range estimation Furthermore, many detected individuals were not residents thus resulting in potentially biased estimates for the parameter in question.

The combined yearly survival probabilities for independent individuals reached 85%. This high value is likely because our estimates come from strictly protected areas where lynx have a higher chance of survival. However, none of the detected individuals used the territory included in the protected areas exclusively, thus potentially exposing themselves to a higher risk. The survival estimated through open population SCR methods in our study site is among the highest reported for lynx. In the Western Carpathians, Dula et al.^50^ conducted an SCR survey on an autochthone lynx population across multiple seasons and found an overall apparent survival (i.e. including emigration) of 63%^50^ with high human-related mortality occurring in the area. The only other values of survival rates available for comparisons come from telemetry studies because almost all of the SCR surveys conducted on lynx in Europe based on camera trapping data used closed population models and thus did not provide information on this parameter. In Switzerland, Breitenmoser-Würsten et al.^5^ found an overall survival rate of 76% for adults and 53.3% for subadults. In Poland, the survival rate was only 63%^54^, considering subadults and adults combined. In three Scandinavian study sites, Andrén et al.^34^ found lower survival rates for subadults (70, 77 and 71%) than adults (87, 91 and 84%), likely due to lower mortality related to vehicle collision and hunting in adulthood. Comparisons to SCR results are however difficult given the contrasting underlying methodologies. Against our expectations, the survival probabilities were higher for males, though not significantly. This was due to a higher overall number of apparent survival events for males, which consist of the number of consecutive detections over years including gaps during which the animal was alive but not detected. A similar open population SCR study, conducted on a low-density ocelot population in Belize, also showed no significant differences in sex-specific survival probabilities, despite they found probabilities of 0.86 for females and 0.78 for males, respectively^10^. The authors suggested the statistical power was not enough to detect significant differences between sexes in these parameters, although they were able to determine sex for a large number of adult ocelots (*n* = 322). These are probably also the reasons why we could not prove statistically significant differences in per capita recruitment rate between sexes (Table 2).

Camera trapping does not allow assessment of the fates of all disappearing individuals and thus does not provide information on natural mortality. However, our auxiliary findings on dead animals show an increasing number of traffic accidents in recent years (Table 1), indicating that the prevention of lynx vehicle collisions represents an important management action for the population. We could not assess the actual impact of illegal killing since carcasses are seldom found, but considering the high survival of the lynx individuals monitored in the study area, illegal killing seems to play no important role within the study site. However, a high poaching rate in the surroundings of our study area was suggested by a modelling approach, which underlines the importance of the protected areas for the survival of the population^4^. These results are consistent with other areas in Europe e.g. Switzerland^5^, and Poland^54^ were traffic accidents and poaching have been found as the main cause of lynx mortality.

Concerning limitations of the open population SCR models, the movement parameter had poor mixing, which is in line with Gardner et al.^55^, meaning Markov chain Monte Carlo iterations slowly converged to unbiased posterior distributions. This is the reason why the parameter was estimated for both sexes combined and it was necessary to run relatively long chains (100,000 iterations).

We attempted to separate survival from emigration and recruitment from immigration using open population SCR models with a spatially explicit model for activity centre relocation^14^. However, the discrimination ability of these models depends on how accurately the movement model can describe the actual activity centre relocation between primary periods^40^. This would require a larger camera trapping array to detect dispersing movements of large carnivores. We, therefore, could not reliably separate emigration and immigration from survival and recruitment, respectively. However, it is reasonable to assume that true survival and recruitment are at least as high as the value we estimated when the underestimation due to the inclusion of emigration and immigration is considered.

### Reproductive parameters and age distribution

Generation time was calculated as the mean age of resident reproducing females at their first documented reproduction, potentially resulting in overestimated values if previous litters went undetected by camera traps. This may be the case for individuals living close to the boundaries of the monitored area, such as the outlier we detected. In Scandinavia, generation time of lynx was investigated across different environments and ranged from two to more than three years with the highest values in the northern territories due to slower life cycles and a different feeding regime and thus body mass^23^. Similar results were found in other areas of Scandinavia^56^. We were not able to fully assess the proportion of reproductive females because sex was not determined for all detected individuals. However, our mean generation time of 2.64 years fall in the range of the Scandinavian studies. We found a rare case of a reproducing female first reproducing at one year of age (Supplementary Table S3). The first reported case of a 1-year-old female Eurasian lynx breeding in the wild was recently observed in the Bohemian–Bavarian–Austrian population range, outside the study area^57^. The authors hypothesised this could be related to very high prey density or high turnover due to poaching. The average number of recruits of 1.97 (range 1–3) was comparable to that reported in Scandinavia. Gaillard et al.^22^, investigated the number of recruits related to litter size and found higher values for multiparous females (i.e. females with multiple reproduction events) ranging approximately between 0.5 and 2 with a litter size between one and four kittens. However, we were not able to determine litter size through camera trapping.

Regarding age distribution, the maximum age determined with certainty in the study area was 10 years, which was constrained by the duration of the study. In Switzerland, Breitenmoser-Würsten et al.^5^ found individuals 14–15 years of age through telemetry. The exact age can only be determined by camera trapping if an individual was first photographed as a kitten. As such, some of the individuals already classified as independent in the first monitoring session reached higher ages than those reported, even if their exact age remained unknown.

### Relative abundance index

According to the RAI results, roe deer abundance in the German part of the study site remained stable even though they were not hunted since 2012. Contrastingly, the red deer population strongly increased (Fig. 3). As lynx in the study area mainly feed on roe deer and are normally only able to kill red deer calves and subadults, they have a stronger effect on the abundance of roe deer than red deer^58^. The stable RAI for roe deer might therefore indicate a limiting effect of lynx, a result consistent with observations from the same^58^ and other areas^59^.

Concerning red fox, intraguild predation of lynx on this species has already been reported in different areas of Scandinavia^60–62^. The fox RAI shows a decrease with increasing lynx numbers, but the trend is not clear. In the study area, the red fox was found as only 1% of lynx kills^31^ meaning other factors such as food availability and diseases could be driving red fox dynamics. RAI results were not related statistically to SCR models as they could not be used as an individual covariate or as a trap covariate since this aspect of the study was limited to the BFNP only. Therefore the RAI results should be interpreted cautiously. Although RAI can be affected by biases attributed to changes in detection probability^63^, we believe this method well represented the abundance trends of the species in question since the study design was uniform over the years.

### Population development and prospects

For lynx, we found increasing abundance and density, as well as positive population growth rates, high survival probabilities and low per capita recruitment, resulting in a low yearly population turnover. This suggests that lynx is not strongly affected by human-related mortality within the study area. This contrasts with Duľa et al.^50^, who found fluctuating density estimates in the Western Carpathians due to illegal killing and traffic collisions. The comparatively stable development in our study area was accompanied by a relatively constant number of family groups (Table 1), i.e. females accompanied by their kittens, which form the most stable structure of the population and are therefore of particular importance. On the other hand, the number of individuals belonging to the least stable part of the population, i.e. floaters likely dispersing through the study area, highly fluctuated throughout the study period (from a minimum of 0 in 2013 to a maximum of 10 in 2017). Furthermore, the increase (or decrease) of the total number of detected individuals from one monitoring season to the following mainly corresponded with an increase (or decrease, respectively) in the number of detected floaters (Table 1). This suggests that the number of floaters visiting the study area in the given year played a major role in determining local abundance lynx for that year. Among all floaters detected in this study, only a lower proportion of individuals originated inside the study area (~ 33%). This reveals potential changes in the Bohemian–Bavarian–Austrian lynx population. Based on coordinated transboundary camera trapping within an area of 13,000 km^2^, the estimated entire population size showed a positive trend and confirmed lynx presence and regular reproduction in a large part of the entire population range, including its outskirts^29^. Thus, fluctuations in the number of floaters may be related to what takes place outside of the protected study area. In particular, increases in the number of floaters reaching the study area from 1 year to the following may be related to a local increase in survival of juveniles and yearlings in part of the Bohemian–Bavarian–Austrian population range. This may be partially related to a local decrease in poaching, possibly as a result of law enforcement and long-term public relations campaigns aimed at improving acceptance in the wake of high-profile poaching incidents. These findings suggest that the overall conditions of the Bohemian–Bavarian–Austrian population have at least partially improved in recent years and this do not only concern the protected areas lying within this population’s range. This is fundamental for the long term genetic viability of a potential future Central European metapopulation as the conservation of species with large spatial requirements cannot rely on protected areas alone^64^. Since data on poaching are almost absent, the causes leading to this positive trend of the population remain speculation. However, other potential explanations such as immigration from other populations or variability in reproduction rates within our study area can be excluded since the Bohemian–Bavarian–Austrian is an isolated population^17^.

## Conclusions

High survival probabilities and regular reproduction thanks to local protection indicate that the protected areas act as a stable source of lynx for the wider distribution range of the Bohemian–Bavarian–Austrian population, which is in line with previous studies^17^. Thus our results show the high value of the protected areas for the persistence of the Bohemian–Bavarian–Austrian As proposed for other large carnivores^18^, we conclude that protected areas can be considered as strategic areas for lynx recovery across its range because full protection increases survival probabilities and ensure stable reproduction. However, we must consider that the management of the surrounding landscapes is fundamental for the long term survival of the population, as protected areas in Central Europe are too small to encompass enough animals to maintain healthy populations over the long term.

Our study revealed crucial demographic parameters of a lynx population that can be used for improving conservation and management plans. Finally, our results stress the importance of long-term systematic monitoring as a basis for the understanding of the population dynamics of large carnivore populations and recommend the use of open population SCR methods to achieve such aims.

## Supplementary Information

Supplementary Information.

## Data Availability

Data supporting the findings of this study are not included due to conservation concerns. Data may, however, be available from the authors upon reasonable request and with permission of both Administrations of both the BFNP and SNP.
