# Supplementary material for: Demography of a Eurasian lynx (Lynx lynx) population within a strictly protected area in Central Europe
Source: Sci Rep. 2021 Oct 6;11:19868. doi: 10.1038/s41598-021-99337-2 (PMC8494906; doi:10.1038/s41598-021-99337-2)
Supplement: Supplementary file 1 — Supplementary Information. [file 41598_2021_99337_MOESM1_ESM.pdf]

Demography of a Eurasian lynx (Lynx lynx) population within a strictly protected area in Central Europe

Stefano Palmero[1*], Elisa Belotti[2,3], Luděk Bufka[3], Martin Gahbauer[4], Christoph Heibl[4,5], Joe Premier[1,4,6], Kirsten Weingarth-Dachs[5], and Marco Heurich[1,4,7]

[1] Chair of wildlife ecology and wildlife management, Faculty of Environment and Natural Resources, University of Freiburg, Tennenbacher Straße 4, 79106 Freiburg, Germany
[2] Faculty of Forestry and Wood Sciences, Czech University of Life Sciences Prague, Kamýcká 1176, 16521 Prague 6, Czech Republic
[3] Department of Research and Nature Protection, Šumava National Park Administration, Sušická 399, 34192 Kašperské Hory, Czech Republic
[4] Department of Visitor Management and National Park Monitoring, Bavarian Forest National Park
[5] Plant Biodiversity Research, Technische Universität München, Emil-Ramann Straße 2, 85354 Freising-Weihenstephan, Germany
[6] Leibniz Institute for Zoo and Wildlife Research (IZW), Alfred-Kowalke-Str. 17, 10315 Berlin, Germany
[7] Inland Norway University of Applied Science Institute for forest and wildlife management, Campus Evenstad, NO-2480 Koppang, Norway

[*]Corresponding author
E-mail address: st.palmero@gmail.com

Supplementary figures and tables

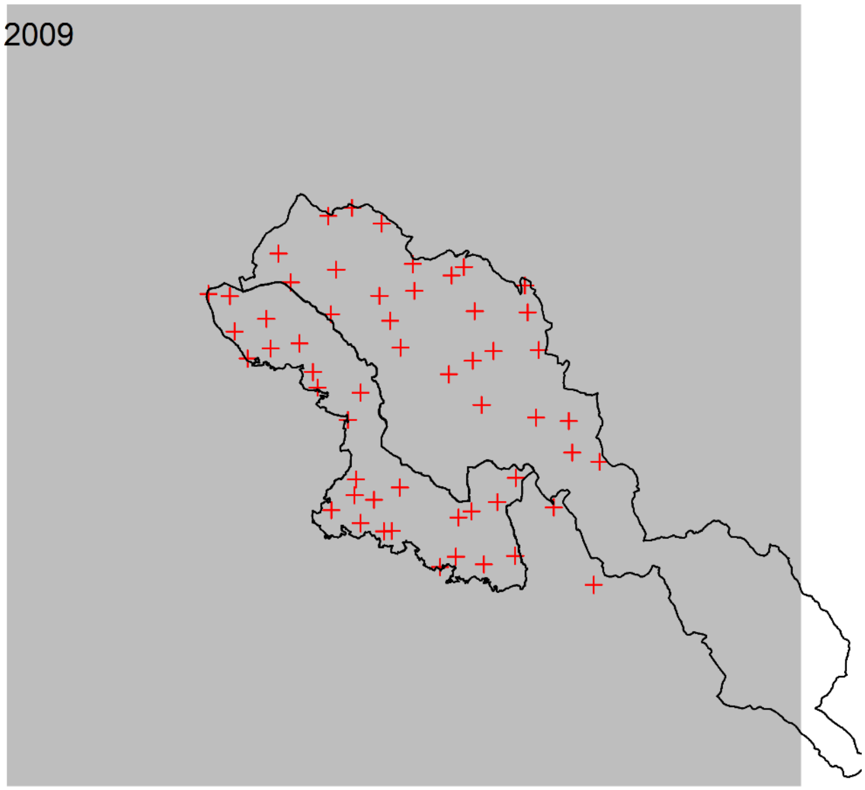

Supplementary Figure S1 Map of the camera trapping sites in the study area in the session 2009 with 18’000 km^2 continuous rectangular buffer used for closed population SCR analysis. The map was created using R 4.0.3 [1] https://www.R-project.org/.

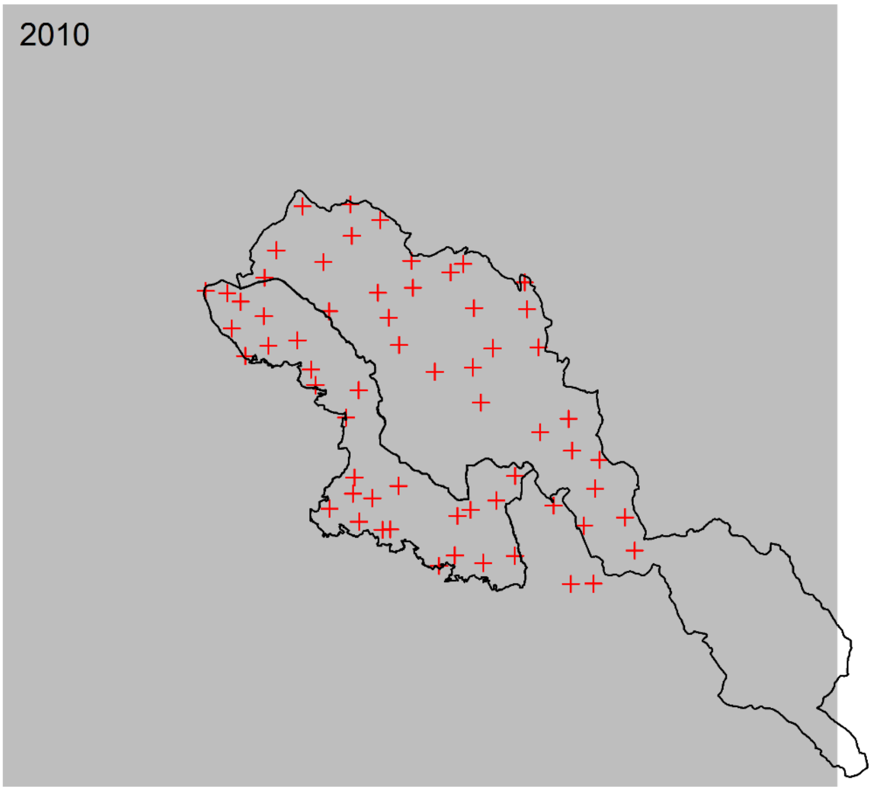

Supplementary Figure S2 Map of the camera trapping sites in the study area in the session 2010 with 18’000 km^2 continuous rectangular buffer used for closed population SCR analysis. The map was created using R 4.0.3 [1] https://www.R-project.org/.

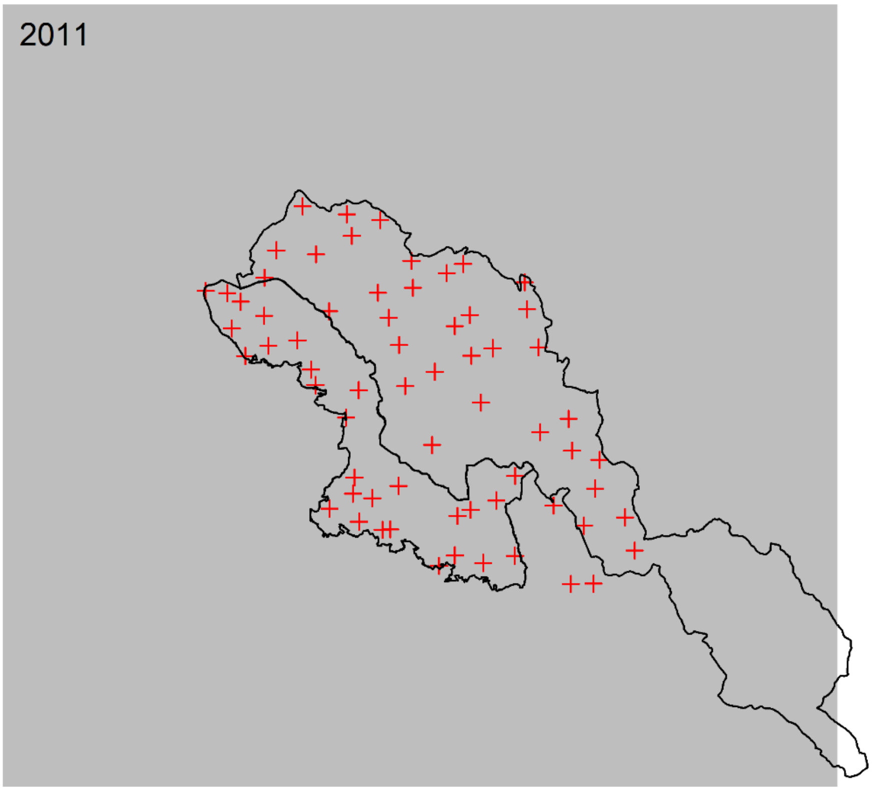

Supplementary Figure S3 Map of the camera trapping sites in the study area in the session 2011 with 18’000 km^2 continuous rectangular buffer used for closed population SCR analysis. The map was created using R 4.0.3 [1] https://www.R-project.org/.

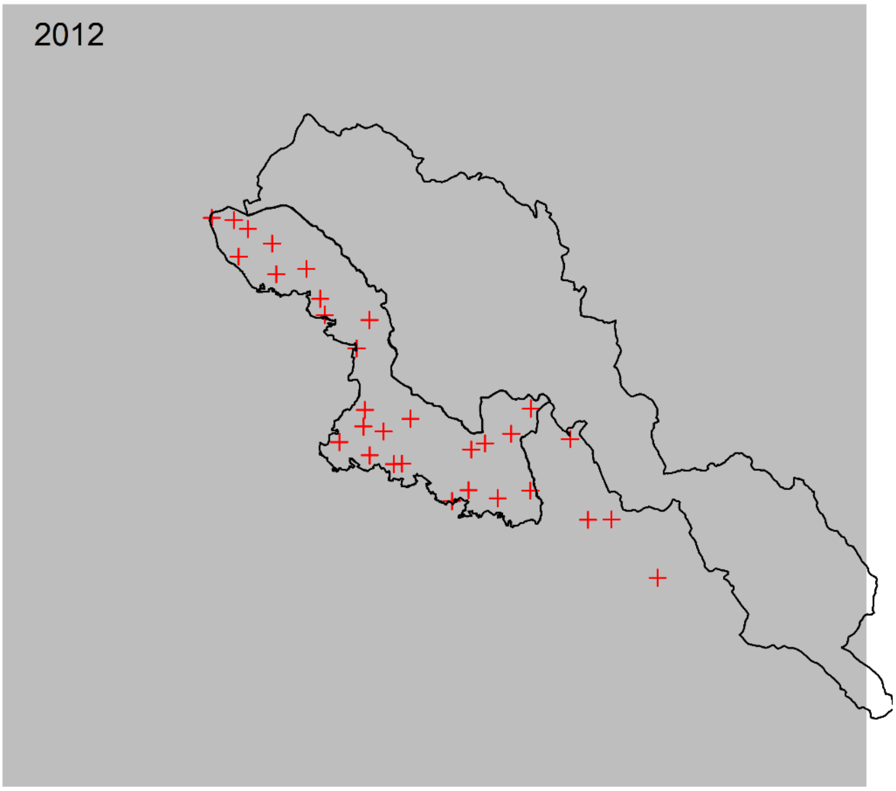

Supplementary Figure S4 Map of the camera trapping sites in the study area in the session 2012 with 18’000 km^2 continuous rectangular buffer used for closed population SCR analysis. In this case, camera traps were not active in the SNP. The map was created using R 4.0.3 [1] https://www.R-project.org/.

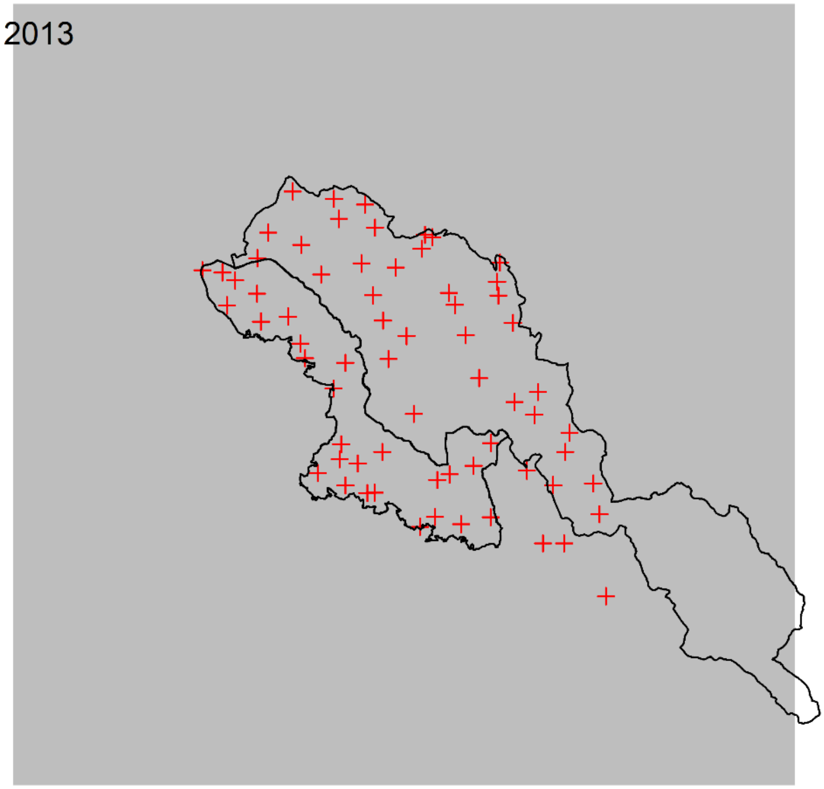

Supplementary Figure S5 Map of the camera trapping sites in the study area in the session 2013 with 18’000 km^2 continuous rectangular buffer used for closed population SCR analysis. The map was created using R 4.0.3 [1] https://www.R-project.org/.

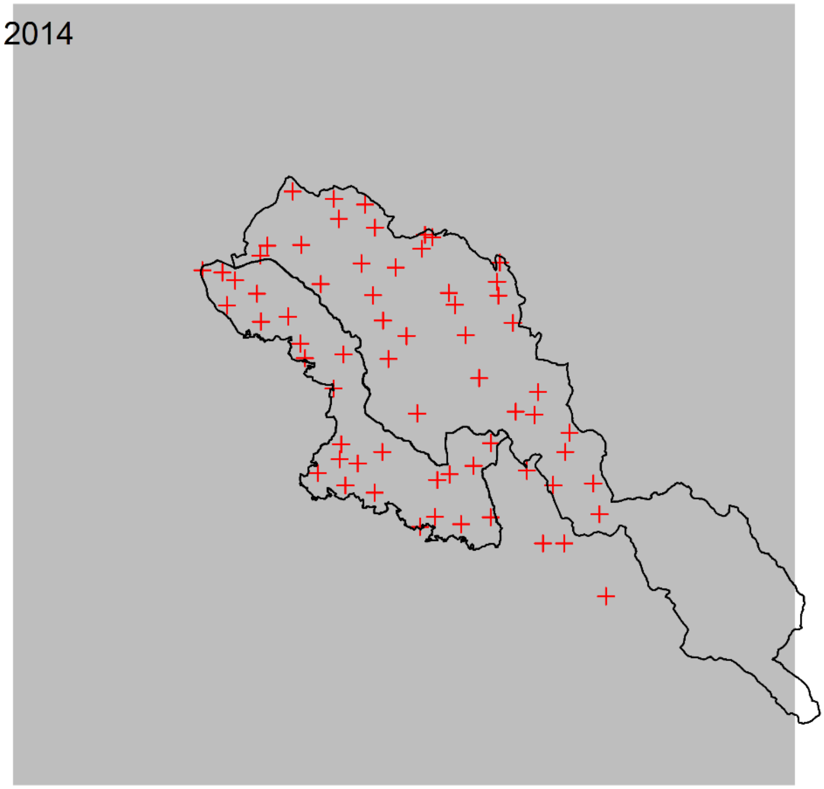

Supplementary Figure S6 Map of the camera trapping sites in the study area in the session 2014 with 18’000 km^2 continuous rectangular buffer used for closed population SCR analysis. The map was created using R 4.0.3 [1] https://www.R-project.org/.

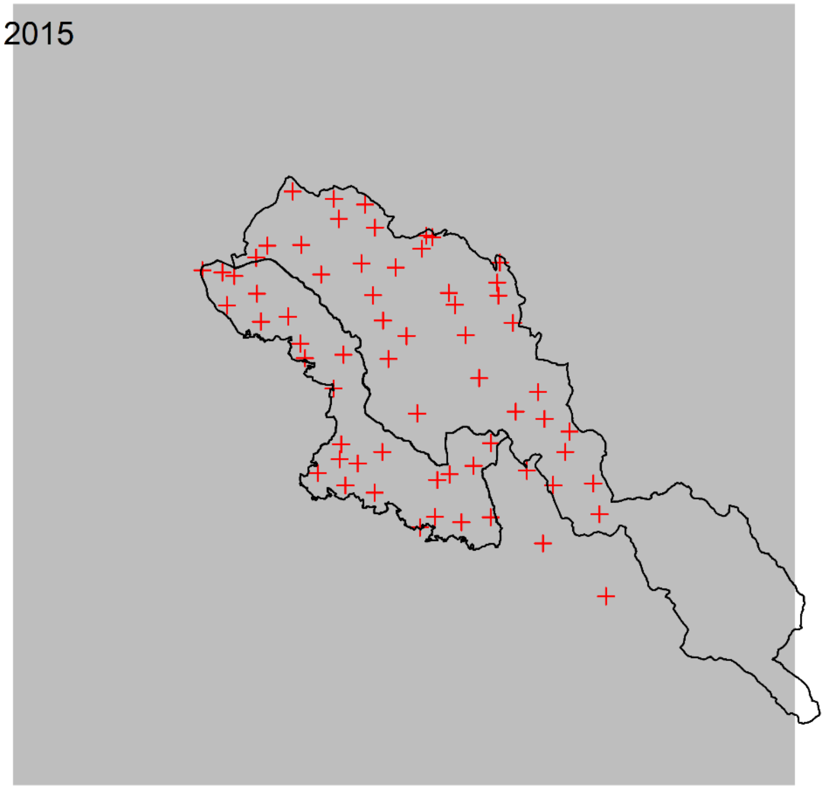

Supplementary Figure S7 Map of the camera trapping sites in the study area in the session 2015 with 18’000 km^2 continuous rectangular buffer used for closed population SCR analysis. The map was created using R 4.0.3 [1] https://www.R-project.org/.

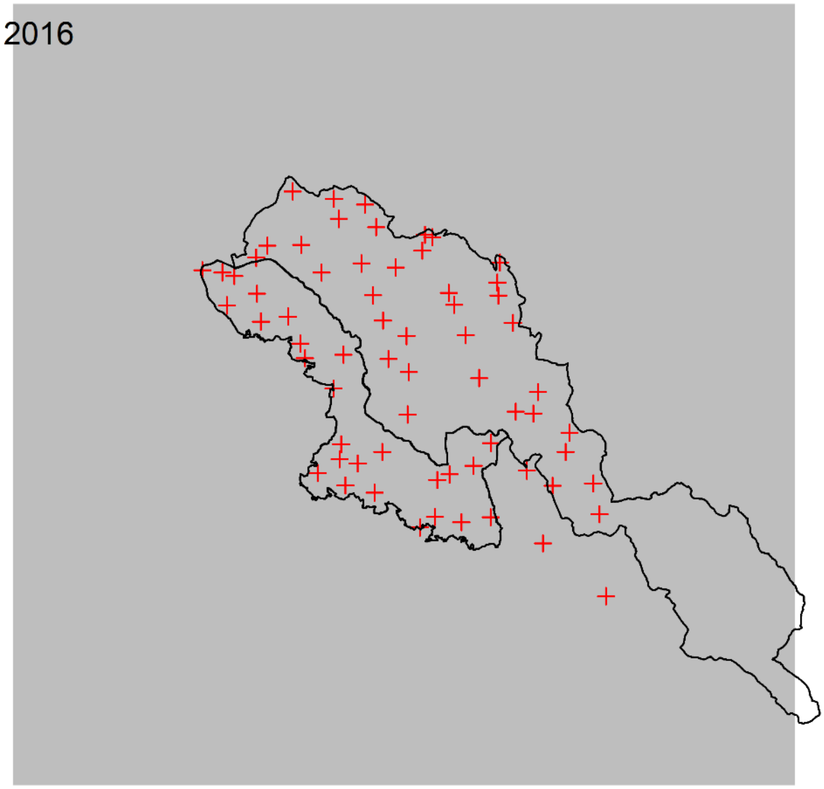

Supplementary Figure S8 Map of the camera trapping sites in the study area in the session 2016 with 18’000 km^2 continuous rectangular buffer used for closed population SCR analysis. The map was created using R 4.0.3 [1] https://www.R-project.org/.

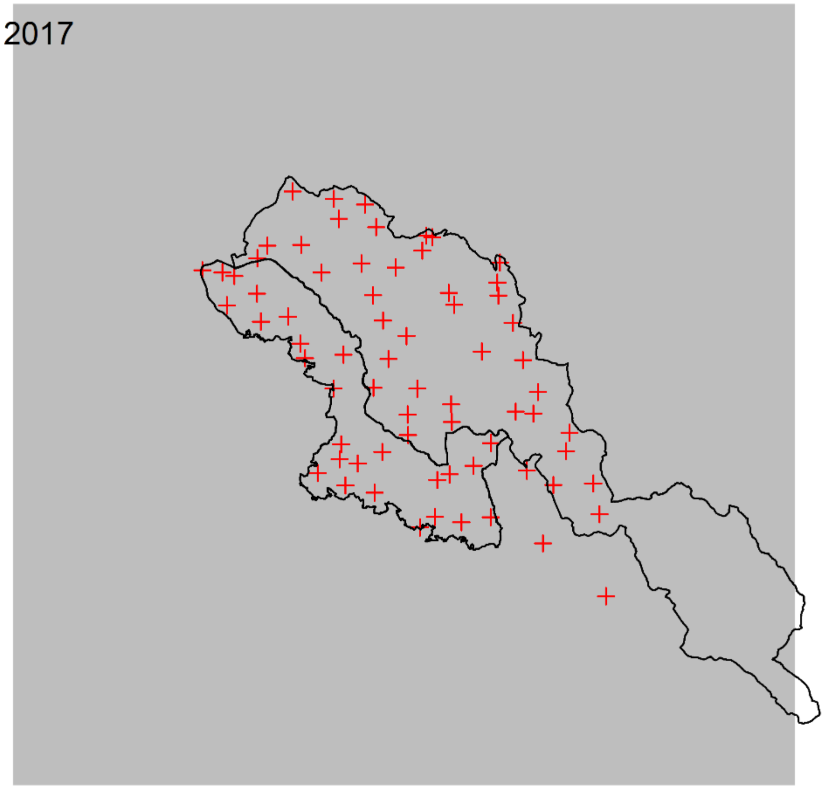

Supplementary Figure S9 Map of the camera trapping sites in the study area in the session 2017 with 18’000 km^2 continuous rectangular buffer used for closed population SCR analysis. The map was created using R 4.0.3 [1] https://www.R-project.org/.

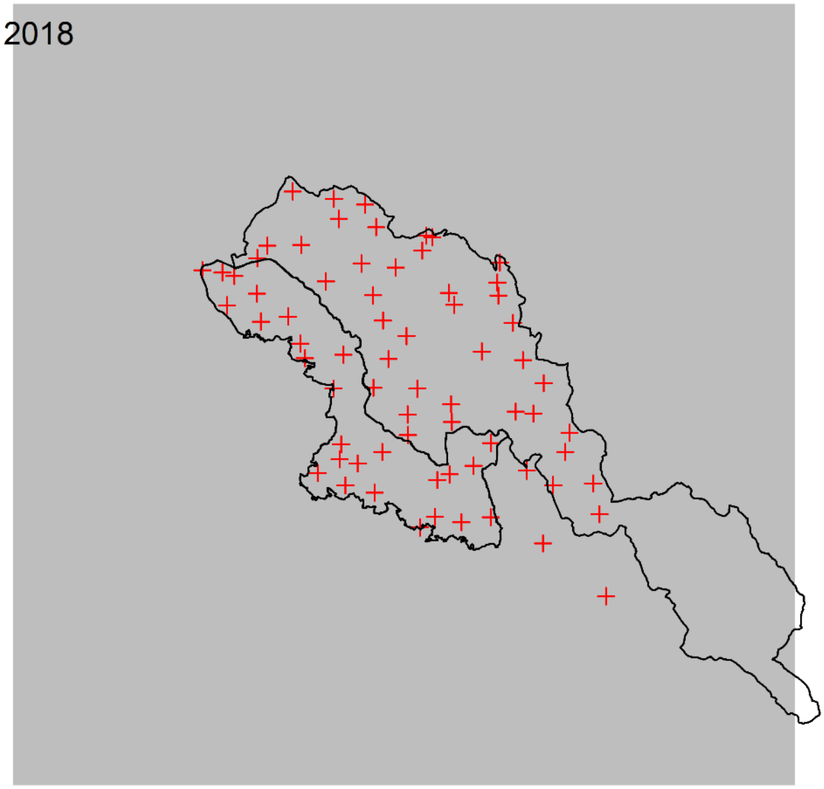

Supplementary Figure S10 Map of the camera trapping sites in the study area in the session 2018 with 18’000 km^2 continuous rectangular buffer used for closed population SCR analysis. The map was created using R 4.0.3 [1] https://www.R-project.org/.

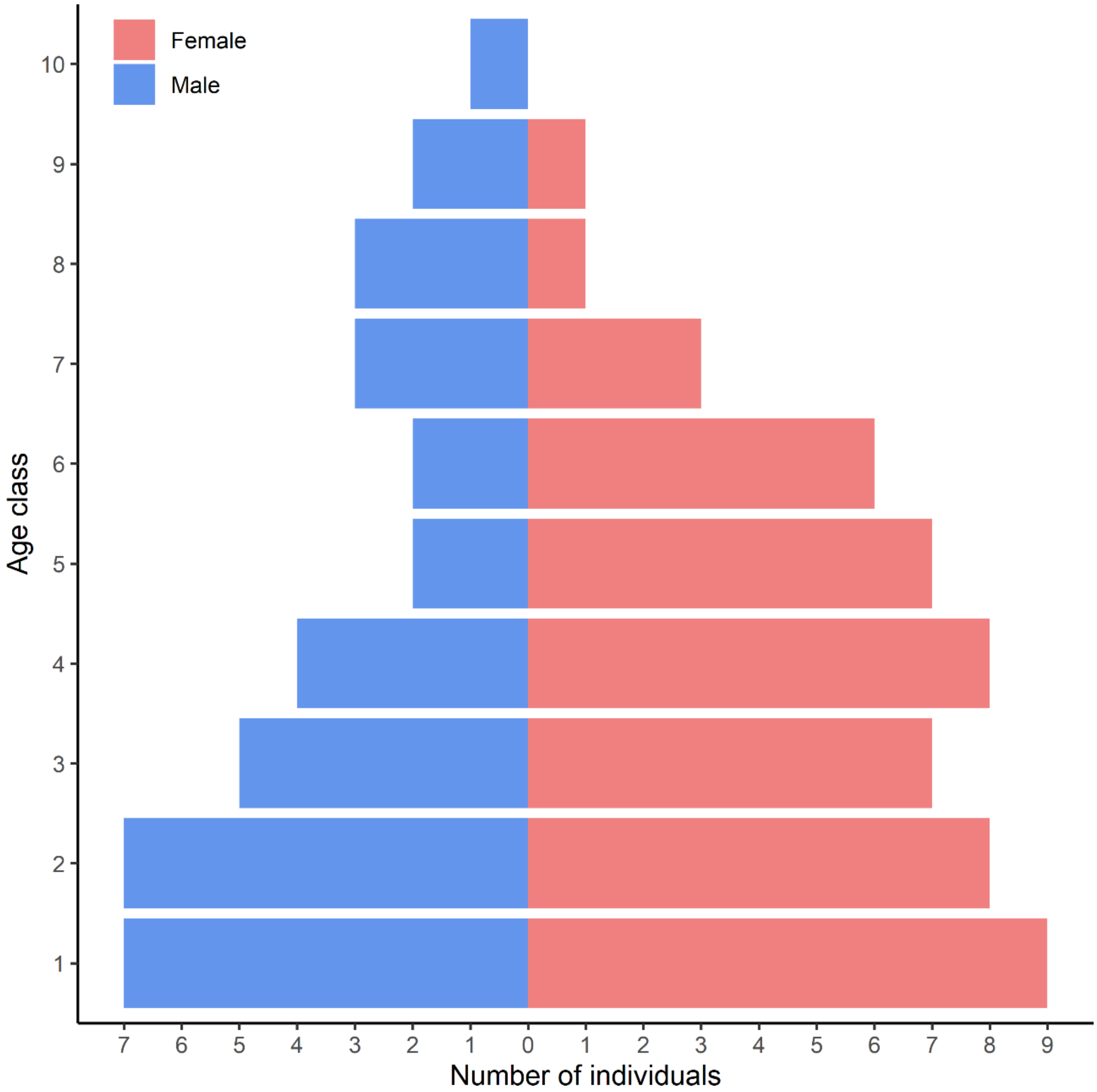

Supplementary Figure S11 Age distribution pyramid of all detected lynx individuals with known age and sex (n = 24 out of 65; 9 males and 15 females). Age classes range from 1 to 10 (i.e. from 1-year-old individuals to 10 years old individuals).

Parameter	Point estimate	Upper 95% CI
lam0M	1.00	1.00
lam0F	1.00	1.01
sigmaM	1.00	1.01
sigmaF	1.01	1.02
gammaM	1.00	1.01
gammaF	1.01	1.02
phiM	1.00	1.01
phiF	1.01	1.02
N1	1.01	1.03
N2	1.02	1.03
N3	1.02	1.03
N4	1.02	1.04
N5	1.02	1.04
N6	1.02	1.04
N7	1.03	1.05
N8	1.03	1.06
N9	1.03	1.06
N10	1.02	1.05
Nm1	1.01	1.01
Nm2	1.01	1.02
Nm3	1.01	1.02
Nm4	1.01	1.02
Nm5	1.01	1.02
Nm6	1.01	1.02
Nm7	1.01	1.02
Nm8	1.01	1.02
Nm9	1.01	1.02
Nm10	1.01	1.02
Nf1	1.01	1.01
Nf2	1.01	1.02
Nf3	1.01	1.02
Nf4	1.01	1.02
Nf5	1.01	1.02
Nf6	1.01	1.02
Nf7	1.01	1.03
Nf8	1.02	1.03
Nf9	1.01	1.03
Nf10	1.01	1.03
sigma_t	1.15	1.30
psex	1.00	1.00
psi	1.01	1.02

Supplementary Table S1 Gelman-Rubin diagnostic statistics of the demographic parameters including point estimates and upper limit of the 95% CI. M and m indicate parameters estimated for males while F and f indicate those estimated for females. The parameter “lam0” refers to baseline detection probability g_0, “sigma” refers to the detection function scale σ, “gamma” refers to per capita recruitment rate, “phi” refers to survival probabilities, “N” refers to abundance, “sigma_t” refers to movement and “psex” indicates the probability of any individual being a female.

Session	Model	ΔAICc	Density ± SE (individuals/100 km²)	95% CI		Realised abundance	95% CI	
				Lcl	Ucl		Lcl	Ucl
2009	D~1; g0~1; sigma~h2; pmix~h2	0.00	1.43 ± 0.91	0.46	4.49	71.05 ± 44.37	26.61	227.67
	D~1; g0~h2; sigma~h2; pmix~h2	19.98	1.27 ± 0.63	0.50	3.18	63.19 ± 31.10	28.04	163.19
2010	D~1; g0~1; sigma~h2; pmix~h2	0.00	1.09 ± 0.35	0.59	2.03	56.32 ± 16.58	33.99	102.40
	D~1; g0~h2; sigma~h2; pmix~h2	2.60	1.12 ± 0.38	0.58	2.15	57.53 ± 18.17	33.63	109.11
2011	D~1; g0~1; sigma~h2; pmix~h2	0.00	0.94 ± 0.26	0.56	1.59	48.58 ± 11.23	32.88	78.86
	D~1; g0~h2; sigma~h2; pmix~h2	4.68	0.93 ± 0.25	0.55	1.57	47.94 ± 11.03	32.53	77.70
2012	D~1; g0~1; sigma~h2; pmix~h2	0.00	2.36 ± 0.79	1.25	4.47	118.41 ± 38.07	66.07	222.37
	D~1; g0~h2; sigma~h2; pmix~h2	4.26	2.42 ± 0.87	1.22	4.81	121.25 ± 42.45	64.58	240.17
2013	D~1; g0~h2; sigma~h2; pmix~h2	0.00	1.12 ± 0.31	0.66	1.90	62.18 ± 15.00	40.83	101.90
	D~1; g0~1; sigma~h2; pmix~h2	3.98	1.07 ± 0.28	0.65	1.78	59.43 ± 13.53	39.91	94.86
2014	D~1; g0~1; sigma~h2; pmix~h2	0.00	1.09 ± 0.27	0.67	1.78	60.58 ± 13.12	41.46	94.64
	D~1; g0~h2; sigma~h2; pmix~h2	2.66	1.09 ± 0.27	0.67	1.77	60.37 ± 13.08	41.32	94.32
2015	D~1; g0~1; sigma~h2; pmix~h2	0.00	1.44 ± 0.32	0.94	2.22	79.86 ± 15.46	56.46	118.67
	D~1; g0~h2; sigma~h2; pmix~h2	3.42	1.44 ± 0.32	0.93	2.22	79.69 ± 15.44	56.34	118.46
2016	D~1; g0~1; sigma~h2; pmix~h2	0.00	1.61 ± 0.32	1.09	2.38	89.01 ± 15.34	65.37	126.84
	D~1; g0~h2; sigma~h2; pmix~h2	3.40	1.61 ± 0.32	1.09	2.38	89.01 ± 15.34	65.37	126.85
2017	D~1; g0~1; sigma~h2; pmix~h2	0.00	1.59 ± 0.32	1.08	2.34	87.83 ± 14.79	65.12	124.45
	D~1; g0~h2; sigma~h2; pmix~h2	3.15	1.59 ± 0.32	1.08	2.34	87.86 ± 14.79	65.14	124.47
2018	D~1; g0~1; sigma~h2; pmix~h2	0.00	1.56 ± 0.32	1.04	2.33	86.32 ± 15.26	62.96	124.15
	D~1; g0~h2; sigma~h2; pmix~h2	0.30	1.57 ± 0.32	1.05	2.34	86.85 ± 15.41	63.23	125.06
	Model average		1.56 ± 0.32	1.05	2.33			

Supplementary Table S2 Estimates and 95% confidence intervals (CI) of closed spatial capture-recapture (SCR) models used for lynx density estimation referring to model formula, ΔAICc, density estimates and realised abundance. The model formula with g0~1, sigma~h2 indicated sex covariate was tested on the detection function scale σ only while the baseline detection probability g_0 was equal for all individuals. The formula with g0~h2, sigma~h2 indicated sex covariate was tested on both the detection parameters

Years	Sex	Detection probability	95% CI		Detection function scale	95% CI		Sex ratio	95% CI	
		Estimate ± SE	Lcl	Ucl	Estimate ± SE	Lcl	Ucl	Estimate ± SE	Lcl	Ucl
2009	F	0.01 ± 0.00	0.00	0.03	2194.50 ± 805.80	1092.99	4406.17	0.67 ± 0.16	0.33	0.89
	M				7559.36 ± 3764.59	3004.92	19016.78	0.33 ± 0.16	0.11	0.67
2010	F	0.02 ± 0.00	0.01	0.03	2385.64 ± 495.70	1594.43	3569.50	0.52 ± 0.14	0.25	0.77
	M				4548.21 ± 537.59	3610.60	5729.31	0.48 ± 0.14	0.23	0.74
2011	F	0.01 ± 0.00	0.00	0.02	4151.50 ± 634.19	3082.65	5590.97	0.53 ± 0.13	0.29	0.76
	M				5184.12 ± 529.30	4246.12	6329.34	0.47 ± 0.13	0.24	0.71
2012	F	0.01 ± 0.00	0.01	0.03	1695.59 ± 295.92	1027.48	2381.01	0.58 ± 0.14	0.30	0.81
	M				3919.39 ± 705.04	2762.59	5560.58	0.42 ± 0.14	0.19	0.69
2013	F	0.00 ± 0.00	0.00	0.00	3535.72 ± 592.81	2551.23	4900.11	0.53 ± 0.13	0.29	0.76
	M	0.01 ± 0.00	0.00	0.01	4047.59 ± 330.15	3450.49	4748.02	0.46 ± 0.13	0.24	0.70
2014	F	0.02 ± 0.00	0.01	0.02	3701.94 ± 300.89	3008.95	4554.53	0.53 ± 0.12	0.30	0.74
	M				3042.82 ± 264.46	2567.05	3606.76	0.47 ± 0.12	0.25	0.70
2015	F	0.02 ± 0.00	0.00	0.02	2710.44 ± 216.39	2318.41	3168.76	0.52 ± 0.11	0.32	0.72
	M				3108.64 ± 191.67	2755.09	3507.55	0.48 ± 0.11	0.28	0.68
2016	F	0.02 ± 0.00	0.01	0.02	3148.27 ± 251.76	2692.22	3681.56	0.52 ± 0.10	0.32	0.71
	M				4015.24 ± 250.37	3553.75	4536.67	0.48 ± 0.10	0.29	0.67
2017	F	0.01 ± 0.00	0.01	0.01	4281.85 ± 402.23	3563.25	5145.38	0.45 ± 0.01	0.27	0.64
	M				4799.01 ± 442.15	4007.68	5746.59	0.55 ± 0.01	0.36	0.73
2018	F	0.01 ± 0.00	0.01	0.02	3705.23 ± 419.60	2969.80	4622.77	0.46 ± 0.10	0.28	0.65
	M	0.02 ± 0.00	0.01	0.02	3514.44 ± 273.27	3018.35	4092.06	0.54 ± 0.10	0.34	0.72

Supplementary Table S3 Estimates and 95% confidence intervals (CI) of closed spatial capture-recapture (SCR) models referring to male (M) and female (F) lynx baseline detection probability *g0*, detection function scale σ (m) and sex ratio.

Name of reproducing females	Age of reproducing females at the first litter	Average litter size
Matylda	3	1.71
Tessa	2	2.00
Hakerl	2	2.00
Luna	2	1.83
Otis	2	1.50
Hope	2	2.00
Shiva	2	2.00
Geli	4	2.50
Misa	3	2.00
Hawei*	7	1.00
Nika	3	2.50
Alina	2	2.50
Olina	2	2.00
Malu	1	2.00
Felis	–	2.00
Kubicka	–	1.50
Nora	–	1.83
Silva	–	2.00
Cherry	–	2.00
Sonea	–	3.00
Zasu	–	1.50
Majka	–	1.50
Mean	2.64	1.97

Supplementary Table S4 Reproductive parameters including generation time and the average number of kittens per reproducing female. Generation time was calculated as the mean of the age of all reproducing females at their first recorded litter while average litter size is the mean of the average number of kittens across all years for each reproducing female. The outlier is indicated by an asterisk (*).

References

1. R Core Team (2020). R: A language and environment for statistical computing. R Foundation for Statistical Computing, Vienna, Austria. URL https://www.R-project.org/.
